# Exponentially Fitted Two-Derivative Runge-Kutta Methods for Simulation of Oscillatory Genetic Regulatory Systems

**DOI:** 10.1155/2015/689137

**Published:** 2015-10-13

**Authors:** Zhaoxia Chen, Juan Li, Ruqiang Zhang, Xiong You

**Affiliations:** Department of Applied Mathematics, Nanjing Agricultural University, Nanjing 210095, China

## Abstract

Oscillation is one of the most important phenomena in the chemical reaction systems in living cells. The general purpose simulation algorithms fail to take into account this special character and produce unsatisfying results. In order to enhance the accuracy of the integrator, the second-order derivative is incorporated in the scheme. The oscillatory feature of the solution is captured by the integrators with an exponential fitting property. Three practical exponentially fitted TDRK (EFTDRK) methods are derived. To test the effectiveness of the new EFTDRK methods, the two-gene system with cross-regulation and the circadian oscillation of the period protein in *Drosophila* are simulated. Each EFTDRK method has the best fitting frequency which minimizes the global error. The numerical results show that the new EFTDRK methods are more accurate and more efficient than their prototype TDRK methods or RK methods of the same order and the traditional exponentially fitted RK method in the literature.

## 1. Introduction

The qualitative analysis and quantitative simulation of gene expression and regulation play an important role in understanding the dynamics of complex processes in cells. Ordinary differential equations (ODEs) have proved to be one of the powerful tools for modeling the complex dynamics of genetic regulation in cells, where the cellular concentrations of mRNAs, proteins, and other molecules are assumed to vary continuously in time (see, e.g., de Jong [1], Widder et al. [2], Polynikis et al. [3], Altinok et al. [4], and Gérard and Goldbeter [5] and the references therein).

Due to the nonlinearity of the ODE models, the closed form of solution is usually not acquirable. Therefore, in order to reveal the dynamics of such gene regulatory systems, one usually resorts to numerical simulation. Up till now, differential equations of gene regulatory systems are mostly simulated by Runge-Kutta (RK) methods, especially by the classical fourth-order Runge-Kutta method, or by the Runge-Kutta-Fehlberg adaptive method (see Butcher [6, 7] and Hairer et al. [8]).

As is often observed in experiments, in a variety of cell processes, genes exhibit an oscillatory behavior. Among examples are sustained oscillations associated with circadian clocks, enzyme synthesis, or the cell cycle (see Goldbeter [9] and Jolley et al. [10]). Unfortunately, when applied to these oscillatory systems, the general purpose RK method often fails to produce satisfactorily efficient numerical results since it did not take into account the special structure of the true solution. There are mainly two deficiencies of the classical RK methods: (i) they cannot produce as accurate numerical results as required even if they have a very high algebraic order and (ii) the true dynamical behavior of the system cannot be preserved as expected in long-term integration.

Recently, some authors have proposed to adapt traditional integrators to problems whose solutions are oscillatory or periodic (see Bettis [11], Gautschi [12], Martín and Ferándiz [13], and Raptis and Simos [14]). Bettis [15] constructed a three-stage method and a four-stage method which can solve the equation *y*′ = *iωy* (*i* ^2^ = −1) exactly. Very recently You [16] developed a new family of phase-fitted and amplification fitted methods of RK type which have been proved very effective for genetic regulatory systems with a limit-cycle structure. You et al. [17] considered a splitting approach for the numerical simulation of genetic regulatory networks with a stable steady state structure. The numerical results of the simulation of a one-gene network, a two-gene network, and a p53-mdm2 network showed that the new splitting methods constructed in this paper are remarkably more effective and more suitable for long-term computation with large steps than the traditional general purpose Runge-Kutta methods.

Motivated by the work of Chan and Tsai [18] and Fang et al. [19] on the two-derivative Runge-Kutta methods (TDRK), the objective of this paper is to develop a novel type of exponentially fitted two-derivative Runge-Kutta (EFTDRK) methods for simulating genetic regulatory systems with an oscillatory structure. These new numerical integrators respect the limit cycle structure of the system and are expected to be more accurate than the traditional RK methods in the long-term integration of gene regulatory systems. In Section 2 we present the main models: one is for gene systems with cross regulations and the other is for the circadian oscillation of the period protein in* Drosophila*. In Section 3, three EFTDRK methods of algebraic order six are constructed. In Section 4 the new EFTDRK methods are applied to the simulation of the two genetic regulatory systems given in Section 2 and their efficiency is compared with that of a sixth-order traditional RK and a sixth-order TDRK method and three exponentially fitted RK methods.  Section 5 is devoted to some conclusive remarks and discussions. The mathematical theory of order conditions and the evaluation of best fitting frequencies for EFTDRK methods are presented in the Appendix.

## 2. Models

### 2.1. Cross-Regulation Systems

An *N*-gene regulatory system can be modeled by a system of ordinary differential equations,

(1) m˙i=−γimit+Rip1t,…,pNt,p˙i=−μipit+Timit,i=1,…,N,

or in matrix form,

(2) m˙t=Rpt−Γmt,p˙t=Tmt−Mpt,

where *m*(*t*) = (*m*_1_(*t*),…, *m*_*N*_(*t*))^*T*^ and *p*(*t*) = (*p*_1_(*t*),…, *p*_*N*_(*t*))^*T*^ are *N*-dimensional vectors which represent the concentrations of mRNAs and proteins at time *t*, respectively, a dot “·” over a variable represents time derivative, *R*(*p*(*t*)) = (*R*_1_(*p*(*t*)),…, *R*_*N*_(*p*(*t*)))^*T*^, *T*(*m*(*t*)) = (*T*_1_(*m*_1_(*t*)),…, *T*_*N*_(*m*_*N*_(*t*)))^*T*^, Γ = diag⁡(*γ*_1_,…, *γ*_*N*_), and *M* = diag⁡(*μ*_1_,…, *μ*_*N*_) are diagonal matrices. For *i* = 1,…, *N*, *m*_*i*_ is the concentration of mRNA *R*_*i*_ produced by gene *G*_*i*_, *p*_*i*_ is the concentration of protein *P*_*i*_ produced by *R*_*i*_, *γ*_*i*_ is the degradation rate of *R*_*i*_, and *μ*_*i*_ is the degradation rate of *P*_*i*_. Function *T*_*i*_(*m*_*i*_) is the translation function. Function *R*_*i*_(*p*(*t*)) is the regulation function, typically defined as sums of functions of *p*_1_,…, *p*_*N*_. If ∂*R*_*i*_/∂*p*_*j*_ > 0, protein *P*_*j*_ is an* activator* of gene *G*_*i*_. If ∂*R*_*i*_/∂*p*_*j*_ < 0, protein *P*_*j*_ is an* inhibitor* of gene *G*_*i*_. If protein *P*_*j*_ has no effect on gene *G*_*i*_, *p*_*j*_ does not appear in *R*_*i*_. Usually, *T*(*m*(*t*)) = (*κ*_1_ *m*_1_(*t*),…, *κ*_*N*_ *m*_*N*_(*t*))^*T*^ = diag⁡(*κ*_1_,…, *κ*_*N*_)*m*(*t*).

In particular, we will be concerned with the following two-gene system:

(3) m˙1=λ1H+p2;θ2,n2−γ1m1,m˙2=λ2H−p1;θ1,n1−γ2m2,p˙1=κ1m1−μ1p1,p˙2=κ2m2−μ2p2,

where *m*_1_ and *m*_2_ are the concentrations of *R*_1_ and *R*_2_, respectively, *p*_1_ and *p*_2_ are the concentrations of the corresponding *P*_1_ and *P*_2_, respectively, *λ*_1_ and *λ*_2_ are the maximal transcription rates of gene 1 and gene 2, respectively, *γ*_1_ and *γ*_2_ are the degradation rates of *R*_1_ and *R*_2_, respectively, *μ*_1_ and *μ*_2_ are the degradation rates of *P*_1_ and *P*_2_, respectively,

(4) H+p2;θ2,n2=p2n2p2n2+θ2n2,H−p1;θ1,n1=θ1n1p1n1+θ1n1

are the* Hill functions* for activation and inhibition, respectively, *n*_1_ and *n*_2_ are the* Hill coefficients*, and *θ*_1_ and *θ*_2_ are the thresholds.

### 2.2. Circadian Rhythms

Another model we are interested in is for circadian oscillations in the period protein (PER) in* Drosophila*. A crucial mechanism for oscillations in the model is the negative feedback exerted by nuclear PER on the production of* per* mRNA. This negative feedback will be described by a Hill type equation, where the Hill coefficient *n* represents the degree of cooperativity, and *K*_*I*_ represents the threshold inhibition constant. It is assumed that* per* mRNA is synthesized in the nucleus and immediately transfers to the cytosol, where it accumulates at a maximum rate *v*_*s*_; there it is degraded enzymatically, in a Michaelian manner, at a maximum rate *v*_*m*_. The rate of synthesis of PER is characterized by an apparent first-order rate constant *k*_*s*_. PER experiences a series of phosphorylations (Edery et al. [20]). For simplicity, it is assumed that there are three states of the protein: unphosphorylated, monophosphorylated, and bisphosphorylated. Goldbeter [21] formulated the five-variable system of equations as follows:

(5) dMdt=vsKInKIn+PNn−vmMKm1+M,dP0dt=ksM−V1P0K1+P0+V2P1K2+P1,dP1dt=V1P0K1+P0−V2P1K2+P1−V3P1K3+P1+V4P2K4+P2,dP2dt=V3P1K3+P1−V4P2K4+P2−k1P2+k2PN−vdP2Kd+P2,dPNdt=k1P2−k2PN,

where the dependent variables *M*, *P*_0_, *P*_1_, *P*_2_, and *P*_*N*_ are the concentrations of cytosolic* per* mRNA, unphosphorylated PER, monophosphorylated PER, bisphosphorylated PER, and nuclear PER, respectively, *V*_*i*_ and *K*_*i*_ (*i* = 1,…, 4) denote the maximum rate and Michaelis constant of the kinase(s) and phosphatase(s) involved in the reversible phosphorylation of *P*_0_ into *P*_1_ and of *P*_1_ into *P*_2_, respectively.

## 3. Methods

### 3.1. Modified Two-Derivative Runge-Kutta Methods

We begin by considering the general initial value problem (IVP) of ordinary differential equations

(6) y˙=fy,y0=y0,

where *y* : [0, +*∞*) → *ℝ* ^*d*^, “$\dot{y}$” represents the first derivative of *y* with respect to time, and *f* : *ℝ* ^*d*^ → *ℝ* ^*d*^ is a sufficiently smooth function. Based on experimental observation of oscillatory behavior in genetic regulatory systems, it is reasonable to make the following assumptions on system (6):

(i) System (6) has a steady state *y* ^*∗*^; that is, *f*(*y* ^*∗*^) = 0.
(ii) System (6) has oscillatory solution near *y* ^*∗*^; that is, the Jacobian *J* = *f*′(*y* ^*∗*^) has at least a pair of complex eigenvalues with nonzero imaginary part.

A special form of* two-derivative Runge-Kutta (TDRK) method* reads

(7) Yi=yn+cihfyn+h2∑j=1saijgYj,i=1,…,s,yn+1=yn+hfyn+h2∑i=1sbigYi,

where *a*_*ij*_, *b*_*i*_, *c*_*i*_, *i*, *j* = 1,…, *s* are real numbers and *g*(*y*)≔*f*′(*y*)*f*(*y*). The order conditions for TDRK methods can be found in Chan and Tsai [18].

In applications, for some choice of the parameter values, system (2) has oscillatory solutions. This motivates us to consider the* modified two-derivative Runge-Kutta (TDRK) methods*

(8) Yi=yn+cihfyn+h2∑j=1saijgYj,i=1,…,s,yn+1=ηνyn+hβνfyn+h2∑i=1sbiνgYi,

where *a*_*ij*_, *i*, *j* = 1,…, *s* are constants and the coefficients *η*(*ν*), *β*(*ν*), *b*_*i*_(*ν*), *i* = 1,…, *s* are the functions of *ν* = *hω* with *h* being the step size and *ω* being the principal frequency of the problem. We assume that *η*(0) = *β*(0) = 1 so that as *ω* → 0 (*ν* → 0) the modified TDRK method (8) reduces to a traditional special TDRK method called the* limit method* or the* prototype method* of (8).

In Kronecker's notation, scheme (8) can be written compactly as

(9) Y=e⊗yn+hc⊗fyn+h2A⊗IdgY,yn+1=ηνyn+hβνfyn+h2bνT⊗IdgY,

where

(10) e=1,1,…,1T,Y=Y1T,Y2T,…,YsTT,gY=gY1T,…,gYsTT,c=c1,…,csT

and *I*_*d*_ is *d* × *d* identity matrix. The TDRK method (7) can be briefly expressed by the following Butcher tableau of coefficients:

(11) $$\begin{array}{c} \;\begin{array}{ccc} e & c & A\\ \eta \left(\nu \right) & \beta \left(\nu \right) & b^{T} \end{array}=\;\begin{array}{ccccc} 1 & c_{1} & a_{11} & \ldots & a_{1s}\\ \vdots & \vdots & \vdots & \vdots & \vdots \\ 1 & c_{s} & a_{s1} & \ldots & a_{ss}\\ \eta \left(\nu \right) & \beta \left(\nu \right) & b_{1} & \ldots & b_{s}. \end{array} \end{array}$$

The order conditions for modified TDRK methods will be derivative via the theory of biordered trees in Appendix. For purpose of construction of practical methods, we list the sixth-order conditions as follows:

(12) ην=1+Oν7,βν=1+Oν6,bTνe=12+Oν5,bTνc=16+Oν4,bTνc2=112+Oν3,bTνAe=124+Oν3,bTνc3=120+Oν2,bTνc·Ae=140+Oν2,bTνAc=1120+Oν2,bTνc4=130+Oν,bTνc2·Ae=160+Oν,bTνc·Ac=190+Oν,bTνAe·Ae=1120+Oν,bTνAc2=1360+Oν,bTνA2e=1720+Oν,

where *c* ^2^ = (0, *c*_2_^2^,…, *c*_*s*_^2^),  *c* ^3^ = (0, *c*_2_^3^,…, *c*_*s*_^3^), *c* ^4^ = (0, *c*_2_^4^,…, *c*_*s*_^4^), and a dot “·” between two vectors indicates a pairwise multiplication. If we assume that

(13) Ae=12c2,  that  is,∑j=1i−1aij=12ci2,i=2,…,s,

then conditions (12) become

(14) ην=1+Oν7,βν=1+Oν6,bTνe=12+Oν5,bTνc=16+Oν4,bTνc2=112+Oν3,bTνc3=120+Oν2,bTνAc=1120+Oν2,bTνc4=130+Oν,bTνc·Ac=1180+Oν,bTνAc2=1360+Oν.

### 3.2. Exponentially Fitted Two-Derivative Runge-Kutta Methods

With the modified TDRK method (8) we associate a linear operator *ℒ* on *C* ^2^[0, *∞*), the linear space of functions on [0, *∞*) with continuous second derivatives, defined by

(15) Lyt=yt+h−ηνyt−hβνy˙t−h2∑i=1sbiνy¨t+cih.

**Definition 1 .**

The modified TDRK method (8) is said to be* exponentially fitted* if the linear operator *ℒ* satisfies

(16) $$\begin{array}{c} L\left[\;y\;\right]\left(\;t\;\right)\equiv 0 \end{array}$$

for the function *y*(*t*) in the reference set

(17) F=tjkexp⁡±λkt,  jk=0,1,…,Mk,  k=0,1,…,N,

where *λ*_*k*_, *k* = 0,1,…, *N* are real or complex numbers and *N*, *M*_*k*_ are nonnegative integers.

In this subsection, we consider four-stage explicit modified TDRK methods given by the following tableau:

(18) $$\begin{array}{c} \;\begin{array}{cccccc} 1 & 0 & & & & \\ 1 & \frac{1}{4} & \frac{1}{32} & & & \\ 1 & \frac{2}{3} & -\frac{2}{81} & \frac{20}{81} & & \\ 1 & 1 & \frac{5}{4} & -\frac{6}{5} & \frac{9}{20} & \\ \eta \left(\nu \right) & \beta \left(\nu \right) & b_{1}\left(\nu \right) & b_{2}\left(\nu \right) & b_{3}\left(\nu \right) & b_{4}\left(\nu \right). \end{array} \end{array}$$

The coefficients *η*(*ν*), *β*(*ν*), *b*_*i*_(*ν*), *i* = 1,2, 3,4 will be obtained by the exponential fitting conditions for some specific reference sets.

#### 3.2.1. First EFTDRK Method

We take the reference set

(19) $$\begin{array}{c} F_{a}=\left\{\;\exp\left(\;\pm i\omega t\;\right),\;\;t\exp\left(\;\pm i\omega t\;\right)\;\right\} \end{array}$$

and assume that the linear operator *ℒ* in (15) vanishes for all functions in *𝔉*_*a*_. This leads to

(20) b2νsin⁡c2ν+b3νsin⁡c3ν+b4sin⁡c4ν=νβν−sin⁡νν2,b1ν+b2νcos⁡c2ν+b3νcos⁡c3ν+b4ν·cos⁡c4ν=ην−cos⁡νν2,ν2b2νc2cos⁡c2ν+b3νc3cos⁡c3ν+b4νc4cos⁡c4ν+2νb2νsin⁡c2ν+b3νsin⁡c3ν+b4νsin⁡c4ν=βν−cos⁡ν,2νb1ν+b2νcos⁡c2ν+b3νcos⁡c3ν+b4νcos⁡c4ν−ν2b2νc2sin⁡c2ν+b3νc3sin⁡c3ν+b4νc4sin⁡c4ν=sin⁡ν.

The third and fourth conditions in (14) for *s* = 4 with higher order terms neglected give

(21) b1ν+b2ν+b3ν+b4ν=12,b2c2ν+b3νc3+b4νc4=16.

We can solve system (20)-(21) for *η*(*ν*), *β*(*ν*), and *b*_*i*_(*ν*), *i* = 1,2, 3,4, whose expressions are extremely complicated. For small values of |*ν*|, we have their Taylor series used as follows:

(22) ην=1+23ν8348364800−2087ν10222953472000+⋯,βν=1+ν61209600+1637ν820901888000−3444061ν103973030871040000+⋯,b1ν=340+ν211200−4601ν4870912000−36863647ν6165542952960000+248988432157ν886770994223513600000+⋯,b2ν=64225−ν25250+5407ν4816480000+52884329ν6155196518400000−391939117499ν881347807084544000000+⋯,b3ν=27200+9ν256000+17ν417920000−9073027ν691968307200000+35868256579ν816068702633984000000+⋯,b4ν=1180−ν216800−427ν4186624000−4822283ν6248314429440000−36912938527ν8130156491335270400000+⋯.

It is easily verified that these coefficients satisfy all conditions in (14). Therefore the method has algebraic order six and we denote this method as EFTDRK4s6a.

#### 3.2.2. Second EFTDRK Method

We take the reference set

(23) $$\begin{array}{c} F_{b}=\left\{\;1,t,t^{2},t^{3},\exp\left(\;i\omega t\;\right),\exp\left(\;2i\omega t\;\right)\;\right\} \end{array}$$

and assume that the linear operator (15) vanishes for all functions in *𝔉*_*b*_. Then we obtain the expression of *η*(*ν*), *β*(*ν*), and *b*_*i*_(*ν*), *i* = 1,2, 3,4.

For small values of |*ν*|, the following Taylor series should be used:

(24) ην=1+299ν8136080000−16561ν10108864000000+⋯,βν=1+ν6189000+12749ν88164800000+9829099ν10277136640000000+⋯,b1ν=340+ν24000−159449ν45443200000−328547263ν6117573120000000−168770118283ν83386105856000000000+⋯,b2ν=64225−ν21875+155713ν45103000000+398361581ν6110224800000000+1477535874781ν834919216640000000000+⋯,b3ν=27200+9ν220000+2153ν4112000000+16429997ν665318400000000+374690334049ν86897623040000000000+⋯,b4ν=1180−ν26000−166921ν48164800000−188918627ν6176359680000000−2614342153777ν855870746624000000000+⋯.

It is easily verified that these coefficients satisfy all conditions in (14). Therefore the method has algebraic order six and we denote this method as EFTDRK4s6b.

#### 3.2.3. Third EFTDRK Method

We take the reference set

(25) $$\begin{array}{c} F_{c}=\left\{\;1,t,t^{2},t^{3},t\exp\left(\;2i\omega t\;\right)\;\right\}, \end{array}$$

and we assume that the linear operator (15) vanishes for all functions in *𝔉*_*c*_. Then we obtain the expression of *η*(*ν*), *β*(*ν*), and *b*_*i*_(*ν*), *i* = 1,2, 3,4. For small values of |*ν*|, the following Taylor series should be used:

(26) ην=1+23ν81360800−2087ν10217728000+⋯,βν=1+ν618900+1637ν881648000−3444061ν103879912960000+⋯,b1ν=340+ν22800−4601ν454432000−36863647ν62586608640000+248988432157ν8338949196185600000+⋯,b2ν=64225−2ν22625+5407ν451030000+52884329ν62424945600000−391939117499ν8317764871424000000+⋯,b3ν=27200+9ν214000+17ν41120000−9073027ν61437004800000+35868256579ν862768369664000000+⋯,b4ν=1180−ν24200−427ν411664000−4822283ν63879912960000−36912938527ν8508423794278400000+⋯.

We denote this method as EFTDRK4s6c.

It is noted that as *ν* → 0, the new methods EFTDRK4s6a, EFTDRK4s6b, and EFTDRK4s6c reduce to a traditional TDRK method given by the following tableau:

(27) $$\begin{array}{c} \;\begin{array}{cccccc} 1 & 0 & & & & \\ 1 & \frac{1}{4} & \frac{1}{32} & & & \\ 1 & \frac{2}{3} & -\frac{2}{81} & \frac{20}{81} & & \\ 1 & 1 & \frac{5}{4} & -\frac{6}{5} & \frac{9}{20} & \\ & & \frac{3}{40} & \frac{64}{225} & \frac{27}{200} & \frac{1}{180}. \end{array} \end{array}$$

## 4. Results

In order to examine the effectiveness of the EFTDRK methods proposed in this paper, we apply these methods as well as a sixth-order RK method and a sixth-order TDRK method to the two genetic regulatory systems presented in Section 2. The numerical methods we will use are listed as follows:

- RK6: the classical RK method of order six presented in [8].
- TDRK4s6: the classical TDRK method of order six presented in [18].
- EFTDRK4s6a, EFTDRK4s6b, EFTDRK4s6c: the three four-stage EFTDRK methods of order six derived in Section 3 of this paper.
- ETFRK4: the fourth-order exponentially and trigonometrically fitted RK method constructed by Simos [22].
- EFRK4: the fourth-order exponentially fitted RK method constructed by Vanden Berghe et al. [23].
- MRK4: the fourth-order modified RK method constructed by Van de Vyver [24].

We will compare the efficiency of these methods by plotting the decimal logarithm of the maximal global error against the computational effort measured by the number of function evaluations.

### 4.1. The Two-Gene System

Denote *y* = (*m*_1_, *m*_2_, *p*_1_, *p*_2_)^*T*^. The Jacobian of system (3) is given by

(28) f′y=−γ100λ1n2θ2n2p2n2−1θ2n2+p2n220−γ2−λ2n1θ1n1p1n1−1θ1n1+p1n120κ10−μ100κ20−μ2

and the function

(29) $$\begin{array}{c} g\left(\;y\;\right)=f^{\prime }\left(\;y\;\right)f\left(\;y\;\right)=\left(\begin{array}{c} \gamma_{1}\left(\gamma_{1}m_{1}-\frac{\lambda_{1}p_{2}^{n_{2}}}{\theta_{2}^{n_{2}}+p_{2}^{n_{2}}}\right)+\frac{\lambda_{1}n_{2}\theta_{2}^{n_{2}}p_{2}^{n_{2}-1}\left(\kappa_{2}m_{2}-\mu_{2}p_{2}\right)}{{\left(\theta_{2}^{n_{2}}+p_{2}^{n_{2}}\right)}^{2}}\\ \gamma_{2}\left(\gamma_{2}m_{2}-\frac{\lambda_{2}\theta_{1}^{n_{1}}}{\theta_{1}^{n_{1}}+p_{1}^{n_{1}}}\right)-\frac{\lambda_{2}n_{1}\theta_{1}^{n_{1}}p_{1}^{n_{1}-1}\left(\kappa_{1}m_{1}-\mu_{1}p_{1}\right)}{{\left(\theta_{1}^{n_{1}}+p_{1}^{n_{1}}\right)}^{2}}\\ -\mu_{1}\left(\kappa_{1}m_{1}-\mu_{1}p_{1}\right)-\kappa_{1}\left(\gamma_{1}m_{1}-\frac{\lambda_{1}p_{2}^{n_{2}}}{\theta_{2}^{n_{2}}+p_{2}^{n_{2}}}\right)\\ -\mu_{2}\left(\kappa_{2}m_{2}-\mu_{2}p_{2}\right)-\kappa_{2}\left(\gamma_{2}m_{2}-\frac{\lambda_{2}\theta_{1}^{n_{1}}}{\theta_{1}^{n_{1}}+p_{1}^{n_{1}}}\right) \end{array}\right). \end{array}$$

We take the values of parameters as follows (Polynikis et al. [3]):

(30) n1=n2=3,λ1=1.15,λ2=2.35,γ1=γ2=1,κ1=κ2=1,μ1=μ2=1,θ1=θ2=0.21.

We solve system ${\dot{m}}_{1}={\dot{m}}_{2}={\dot{p}}_{1}={\dot{p}}_{2}=0$ by Newton iteration for the unique positive steady state of system (3) which is given by

(31) m1∗,m2∗,p1∗,p2∗=0.475099,0.186810,0.475099,0.186810.

The Jacobian matrix at the steady state has the eigenvalues

(32) ξ1,2=−2.049997±1.049997i,ξ3,4=0.049997±1.049997i.

**Figure 1:**
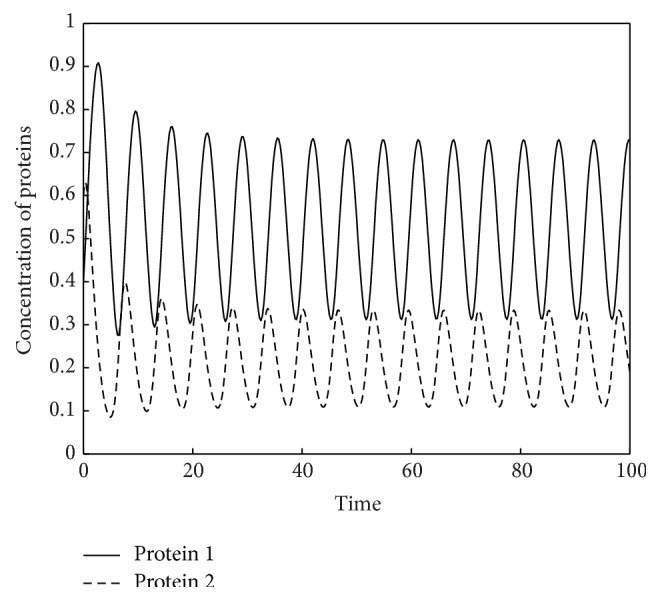
Time evolution of proteins in the two-gene system.

Since these eigenvalues have nonzero imaginary parts, the solution near the steady state is oscillatory with frequency *ω* = 0.989478. This oscillatory behavior of the two proteins is shown in Figure 1 which is plotted straightly by the classical Runge-Kutta method of order four.

For the initial values (*m*_1_(0), *m*_2_(0), *p*_1_(0), *p*_2_(0)) = (0.6,0.8,0.4,0.6) near the equilibrium point, we solve system (3) on the interval [0,100] by the methods EFTDRK4s6a, EFTDRK4s6b, and EFTDRK4s6c with step sizes *h* = 1/2^*i*^, *i* = 2,3, 4,5, with respect to best fitting frequencies *ω*. Tables 1 [Table tab2] [Table tab3]–4 display the global error (GE) of Protein 1 for comparison.

**Figure 2:**
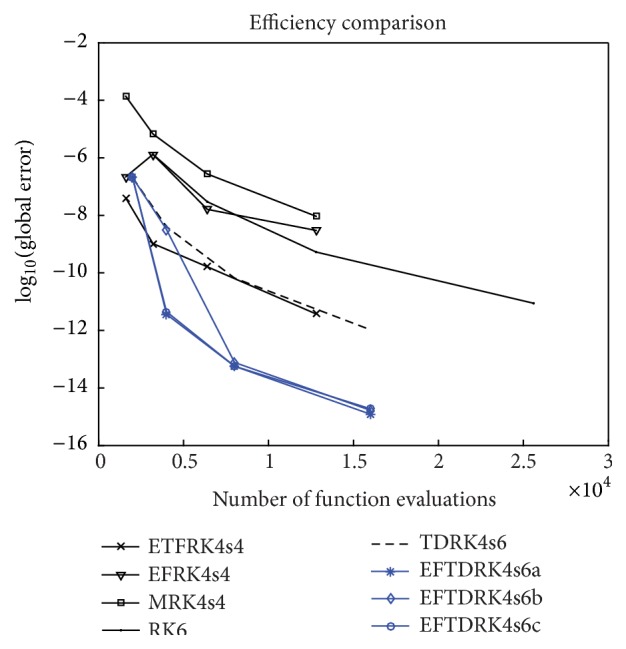
Two-gene system: global error versus function evaluations.

In Figure 2 we compare the efficiency of the eight methods by plotting the global error against the number of evaluations of nonlinear functions *f* and *g*.

### 4.2. PER Oscillations in* Drosophila*

Denote *y* = (*M*, *P*_0_, *P*_1_, *P*_2_, *P*_*N*_)^*T*^. The Jacobian of system (5) is given by
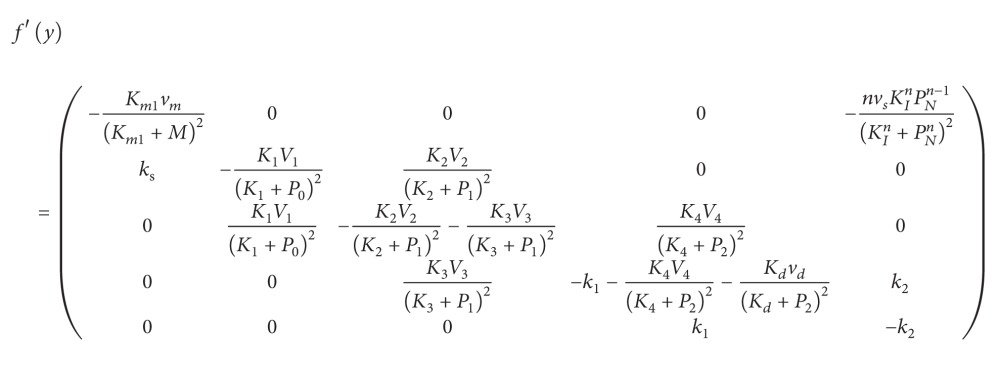
(33)and the function *f*′(*y*)*f*(*y*) = *g*(*y*) = (*g*_1_, *g*_2_, *g*_3_, *g*_4_, *g*_5_)^*T*^ with

(34) g1=nvsKInPNn−1−k1P2+k2PNKIn+PNn2+Km1vmMvmKIn+PNn−KInvsKm1+MKIn+PNnKm1+M3,g2=−K1V1ksM−U+BK1+P02+K2V2U−B−C+DK2+P12+ks−MvmKm1+M+KInvsKIn+PNn,g3=K1V1ksM−U+BK1+P02−K2V2K2+P12+K3V3K3+P12U−B−C+D+K4V4−k1P2+k2PN+C−D−EK4+P22,g4=k2k1P2−k2PN+K3V3U−B−C+DK3+P12−k1+K4V4K4+P22+KdvdKd+P22·−k1P2+k2PN+C−D−E,g5=k2−k1P2+k2PN+k1−k1P2+k2PN+C−D−E,

where

(35) U=P0V1K1+P0,B=P1V2K2+P1,C=P1V3K3+P1,D=P2V4K4+P2,E=P2vdKd+P2.

The parameter values in model (5) are given by (Goldbeter [21])

(36) vs=0.76 μM/h,vm=0.65 μM/h,ks=0.38 h−1,vd=0.95 μM/h,k1=1.9 h−1,k2=1.3 h−1,KI=1 μM,Kd=0.2 μM,Km1=0.5 μM,K1=K2=K3=K4=2 μM,n=4,V1=3.2 μM/h,V2=1.58 μM/h,V3=5 μM/h,V4=2.5 μM/h.

System $\dot{M}={\dot{P}}_{0}={\dot{P}}_{1}={\dot{P}}_{2}={\dot{P}}_{N}=0$ is solved for the unique positive steady state

(37) M∗,P0∗,P1∗,P2∗,PN∗=1.851476,1.049558,0.672924,0.570982,0.834512.

Thus the five eigenvalues of the Jacobian matrix at the steady state are

(38) ξ1=−4.266573,ξ2=−1.834793,ξ3,4=0.032824±0.297276i,ξ5=−0.829294.

**Figure 3:**
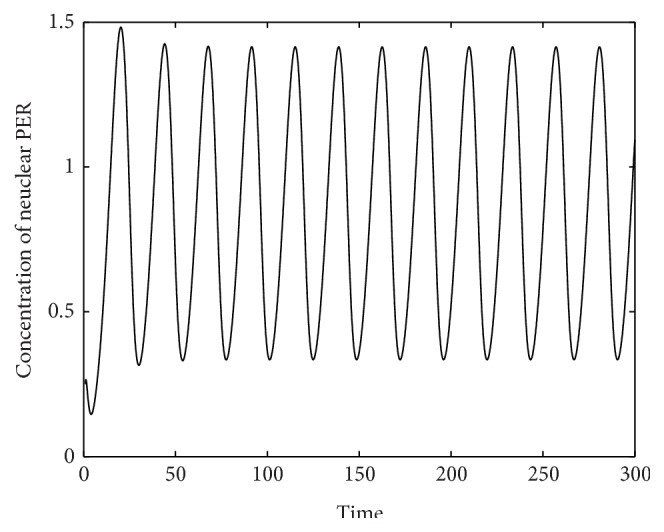
Time evolution of nuclear PER in* Drosophila.*

Since *ξ*_3,4_ have nonzero imaginary parts, the solution near the steady state (*M* ^*∗*^, *P*_0_^*∗*^, *P*_1_^*∗*^, *P*_2_^*∗*^, *P*_*N*_^*∗*^) is oscillatory with frequency *ω* = 0.265113.  Figure 3 displays time evolution of the concentration of the nuclear protein *P*_*N*_.

For the initial values (*M*(0), *P*_0_(0), *P*_1_(0), *P*_2_(0), *P*_*N*_(0)) = (0.1,0.25,0.25,0.25,0.25) near the equilibrium point, we integrate system (3) on the interval [0,100] with step sizes *h* = 1/2^*i*^, *i* = 1,2, 3,4, 5. The numerical results are presented in Tables 5 [Table tab6] [Table tab7] [Table tab8]–9.

**Figure 4:**
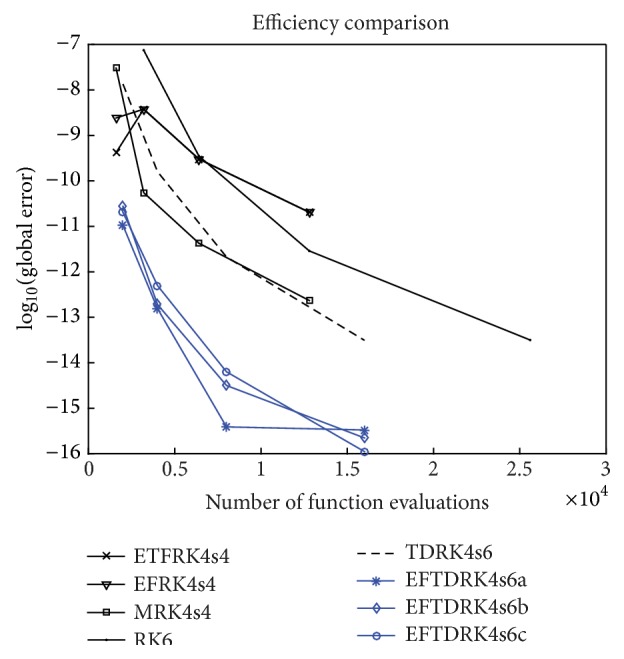
Two-gene system: global error versus function evaluations.

In Figure 4 we plot the efficiency curves for the eight methods.

It can be seen from Tables 1–9 and Figures 2 and 4 that the EFTDRK methods are more efficient than the other methods used for comparison.

## 5. Conclusions and Discussions

Oscillation is frequently observed in all living cells. Whether or not this feature is accurately preserved in simulation has a critical effect on the comprehension of genetic regulation systems. Now that the traditional simulation algorithms perform poorly in simulating the oscillatory genetic regulatory networks, new and more effective simulation technology is called for. In this paper, classical two-derivative Runge-Kutta methods are adapted to the oscillatory character of genetic regulatory systems. The newly developed exponentially fitted two-derivative Runge-Kutta methods (EFTDRK) adopt functions of *ν* = *ωh*, the product of the fitting frequency *ω* and the step size *h*, as weight coefficients in the update. As the fitting frequency tends to zero, EFTDRK methods reduce to their prototype TDRK methods of the same algebraic order.

It should be noted that, in applying an exponentially/trigonometrically fitted method to oscillatory problems, a fitting frequency *ω*, an accurate estimate of the principal frequency, must be obtained in advance. However, for a given oscillatory system, the true frequency is in general not available. In the existing literature, all the methods of fitted type share a common value of the fitting frequency *ω* once it is well estimated. According to the argument in Appendix A.3, for a given differential equation (given function *f*), the global error (GE) of an EFTDRK method (e.g., EFTDRK4s6a) depends on the coefficients (and thus *ν* = *ωh*). If we consider GE as a function *ω*, GE(*ω*) then we take the fitting frequency as the value of *ω* that minimizes the global error. We call it the “best fitting frequency.” It is also noted that it may happen that GE(*ω*) is (much) larger than GE(0). That is, EFTDRK4s6a may be less effective than its prototype TDRK method (TDRK4s6, the case *ω* = 0) if an inappropriate value of the fitting frequency *ω* is employed.

EFTDRK methods have two advantages: they respect the second-order structure of the true solution and they can simulate exactly some standard oscillatory functions such as exp⁡(*iωt*) and exp⁡(2*iωt*). These characteristic properties contribute to their high accuracy and high efficiency. The EFTDRK methods developed in this paper, a category of structure-preserving algorithms, open a new approach to simulating genetic regulatory systems with an oscillation structure.

## Figures and Tables

**Table 1:** Two-gene systems: global error of Protein 1 with step size *h* = 1/4.

	ETFRK4	EFRK4	MRK4	RK6
*ω*	0.659	0.100	0.412	
GE	3.9971*e* − 08	2.1795*e* − 07	1.3827*e* − 04	1.3216*e* − 06

	TDRK4s6	EFTDRK4s6a	EFTDRK4s6b	EFTDRK4s6c

*ω*		0	0	0
GE	2.0703*e* − 07	2.0703*e* − 07	2.0703*e* − 07	2.0703*e* − 07

**Table 2 tab2:** Two-gene systems: global error of Protein 1 with step size *h* = 1/8.

	ETFRK4	EFRK4	MRK4	RK6
*ω*	0.653	1.815	0.640	
GE	1.0441*e* − 09	1.3170*e* − 06	6.7673*e* − 06	2.9632*e* − 08

	TDRK4s6	EFTDRK4s6a	EFTDRK4s6b	EFTDRK4s6c

*ω*		3.145	2.395	1.573
GE	4.0028*e* − 09	3.6251*e* − 12	3.1940*e* − 09	4.4587*e* − 12

**Table 3 tab3:** Two-gene systems: global error of Protein 1 with step size *h* = 1/16.

	ETFRK4	EFRK4	MRK4	RK6
*ω*	0.751	0.022	0.813	
GE	1.6283*e* − 10	1.6144*e* − 08	2.7404*e* − 07	5.3117*e* − 10

	TDRK4s6	EFTDRK4s6a	EFTDRK4s6b	EFTDRK4s6c

*ω*		2.964	2.292	1.482
GE	6.6183*e* − 11	5.6954*e* − 14	7.6938*e* − 14	5.6954*e* − 14

**Table 4:** Two-gene systems: global error of Protein 1 with step size *h* = 1/32.

	ETFRK4	EFRK4	MRK4	RK6
*ω*	1.356	0.011	0.931	
GE	3.7377*e* − 12	2.9958*e* − 09	9.2907*e* − 09	8.7911*e* − 12

	TDRK4s6	EFTDRK4s6a	EFTDRK4s6b	EFTDRK4s6c

*ω*		2.891	2.185	1.445
GE	1.0523*e* − 12	1.2212*e* − 15	1.6653*e* − 15	1.8874*e* − 15

**Table 5:** PER oscillations in *Drosophila*: global error of *P*_*N*_ with step size *h* = 1/2.

	ETFRK4	EFRK4	MRK4	RK6
*ω*	0.811	1.311	0.290	
GE	2.2113*e* − 04	0.3821	0.0011	0.0011

	TDRK4s6	EFTDRK4s6a	EFTDRK4s6b	EFTDRK4s6c

*ω*		1.971	1.369	0.991
GE	1.2580*e* − 06	8.4100*e* − 10	1.1408*e* − 09	1.3104*e* − 09

**Table 6 tab6:** PER oscillations in *Drosophila*: global error of *P*_*N*_ with step size *h* = 1/4.

	ETFRK4	EFRK4	MRK4	RK6
*ω*	0.010	0.002	0	
GE	4.1924*e* − 10	2.4214*e* − 09	3.0138*e* − 08	7.4781*e* − 08

	TDRK4s6	EFTDRK4s6a	EFTDRK4s6b	EFTDRK4s6c

*ω*		1.934	1.363	0.968
GE	1.3468*e* − 08	1.0491*e* − 11	2.7490*e* − 11	2.0292*e* − 11

**Table 7 tab7:** PER oscillations in *Drosophila*: global error of *P*_*N*_ with step size *h* = 1/8.

	ETFRK4	EFRK4	MRK4	RK6
*ω*	0	0	0.047	
GE	3.7713*e* − 09	3.7713*e* − 09	5.5061*e* − 11	3.5174*e* − 10

	TDRK4s6	EFTDRK4s6a	EFTDRK4s6b	EFTDRK4s6c

*ω*		1.895	1.356	0.947
GE	1.5941*e* − 10	1.5565*e* − 13	1.9101*e* − 13	4.9882*e* − 13

**Table 8 tab8:** PER oscillations in *Drosophila*: global error of *P*_*N*_ with step size *h* = 1/16.

	ETFRK4	EFRK4	MRK4	RK6
*ω*	0	0	0.057	
GE	3.0163*e* − 10	3.0163*e* − 10	4.2553*e* − 12	2.8773*e* − 12

	TDRK4s6	EFTDRK4s6a	EFTDRK4s6b	EFTDRK4s6c

*ω*		1.871	1.354	0.935
GE	2.1183*e* − 12	3.8858*e* − 16	3.1641*e* − 15	6.3283*e* − 15

**Table 9:** PER oscillations in *Drosophila*: global error of *P*_*N*_ with step size *h* = 1/32.

	ETFRK4	EFRK4	MRK4	RK6
*ω*	0	0	0.061	
GE	2.0380*e* − 11	2.0380*e* − 11	2.3392*e* − 13	3.1641*e* − 14

	TDRK4s6	EFTDRK4s6a	EFTDRK4s6b	EFTDRK4s6c

*ω*		1.880	1.370	0.931
GE	3.1086*e* − 14	3.3307*e* − 16	0	1.1102*e* − 16

**Table 10:** Trees of order up to order five and the values of corresponding functions.

Tree	*ρ*	*α*	*γ*	Φ_*i*_	*ℱ*
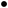	1	1	1		*f*

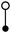	2	1	2	1	*g*

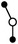	3	1	6	*c*_*i*_	*g*′*f*

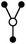	4	1	12	*c*_*i*_^2^	*g*′′(*f*, *f*)
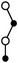	4	1	24	$\underset{j}{\sum }a_{ij}$	*g*_*y*_ *g*

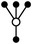	5	1	20	*c*_*i*_^3^	*g* ^(3)^(*f*, *f*, *f*)
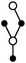	5	3	40	$c_{i}\underset{j}{\sum }a_{ij}$	*g*′′(*f*, *g*)
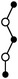	5	1	120	$\underset{j}{\sum }a_{ij}c_{j}$	*g*′*g*

**Table 11:** Trees of order six and the values of corresponding functions.

Tree	*ρ*	*α*	*γ*	Φ_*i*_	*ℱ*
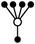	6	1	30	*c*_*i*_^4^	*g* ^(4)^(*f*, *f*, *f*, *f*)

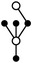	6	6	60	$c_{i}^{2}\underset{j}{\sum }a_{ij}$	*g* ^(3)^(*f*, *f*, *g*)

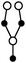	6	3	120	${\left(\underset{j}{\sum }a_{ij}\right)}^{2}$	*g*′′(*g*, *g*)

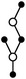	6	4	180	$c_{i}\underset{j}{\sum }a_{ij}c_{j}$	*g*′′(*f*, *g*′*f*)

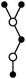	6	1	360	$\underset{j}{\sum }a_{ij}c_{j}^{2}$	*g*′*g*′′(*f*, *f*)

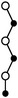	6	1	720	$\underset{j,k}{\sum }a_{ij}a_{jk}$	*g*′*g*′′*g*

## Appendix

### A. Bicolored Trees and Order Conditions for Modified TDRK Methods

#### A.1. Bicolored Trees

In this appendix we present the theory of bicolored rooted trees which forms the basis of the derivation of order conditions for modified TDRK methods.

Definition A.1 A.1. The set BT of bicolored rooted trees is defined recursively as follows:

(i) The graph ∙ and  belong to BT; they are denoted by *τ* and *τ*_2_, respectively. The black vertex in each of the two trees is called the root.
(ii) If *u*_1_,…, *u*_*m*_ ∈ BT, then the tree [*u*_1_,…, *u*_*m*_]_2_ obtained by grafting the roots of *u*_1_,…, *u*_*m*_ onto the white vertex of *τ*_2_.

Definition A.2 A.2. For each tree *t* ∈ BT, the elementary differential *ℱ*(*τ*)(*y*) is a function *ℝ* ^*d*^ → *ℝ* ^*d*^ defined recursively as follows:

(i) *ℱ*(∙)(*y*) = *f*(*y*) and *ℱ*()(*y*) = *g*(*y*).
(ii) For *u* = [*u*_1_,…, *u*_*m*_]_2_ ∈ BT,
  (A.1) $$\begin{array}{c} F\left(\;u\;\right)\left(\;y\;\right)=g^{\left(m\right)}\left(\;F\left(\;u_{1}\;\right)\left(\;y\;\right),\ldots ,F\left(\;u_{m}\;\right)\left(\;y\;\right)\;\right). \end{array}$$

Definition A.3 A.3. The function *ρ* : BT → *ℕ* is defined recursively as follows:

(i) *ρ*(∙) = 1 and *ρ*() = 2.
(ii) For *u* = [*u*_1_,…, *u*_*m*_]_2_ ∈ BT,
  (A.2) $$\begin{array}{c} \rho \left(\;u\;\right)=2+\overset{m}{\underset{i=1}{\sum }}\rho \left(\;u_{i}\;\right). \end{array}$$

For each *u* ∈ BT, *ρ*(*u*) is called the order of the tree *t*. Actually, *ρ*(*u*) is the number of vertices of the tree *u*. The set of trees of order *q* is denoted by BT_*q*_.

Definition A.4 A.4. The function *α* : BT → *ℕ* is defined recursively as follows:

(i) *α*(∙) = 1 and *α*() = 1.
(ii) For *u* = [*u*_1_^*r*_1_^,…, *u*_*m*_^*r*_*m*_^]_2_ ∈ BT,
  (A.3) $$\begin{array}{c} \alpha \left(\;u\;\right)=\left(\;\rho \left(\;u\;\right)-2\;\right)!\overset{m}{\underset{i=1}{\prod }}\frac{1}{r_{i}!}{\left(\frac{\alpha \left(u_{i}\right)}{\rho \left(u_{i}\right)!}\right)}^{r_{i}}, \end{array}$$

where *r*_*i*_ is the multiplicity of *u*_*i*_, *i* = 1,…, *m*.

Definition A.5 A.5. The function *γ* : BT → *ℕ* is defined recursively as follows:

(i) *γ*(∙) = 1 and *γ*() = 2.
(ii) For *u* = [*u*_1_,…, *u*_*m*_]_2_ ∈ BT,
  (A.4) $$\begin{array}{c} \gamma \left(\;u\;\right)=\rho \left(\;u\;\right)\left(\;\rho \left(\;u\;\right)-1\;\right)\overset{m}{\underset{i=1}{\prod }}\gamma \left(\;u_{i}\;\right). \end{array}$$

For each *u* ∈ BT, *γ*(*u*) is called the density of the tree *t*.

Definition A.6 A.6. For the modified TDRK method (8), the elementary weight vector Φ(*t*) = (Φ_*i*_(*t*))_*i*=1_^*s*^ (*t* ∈ BT∖{*τ*}) is defined as follows:

(i) Φ_*i*_() = 1.
(ii) For *u* = [*τ* ^*r*_1_^, *u*_2_,…, *u*_*m*_]_2_ ∈ BT, *ρ*(*u*_*i*_) ≥ 2, *i* = 2,…, *m*,
  (A.5) $$\begin{array}{c} \Phi_{i}\left(\;u\;\right)=c_{i}^{r_{1}}\overset{m}{\underset{i=1}{\sum }}a_{ij}\Phi_{j}\left(\;u_{2}\;\right)\cdots \overset{m}{\underset{i=1}{\sum }}a_{il}\Phi_{l}\left(\;u_{m}\;\right). \end{array}$$

Theorem A.7 A.7. The exact solution *y*(*t*_*n*_ + *h*) and the numerical solution *y*_*n*+1_ produced by the modified TDRK method (8) have the following expansions:

(A.6) ytn+h=yn+hfyn+∑u∈BT∖τhρuρu!αuFuyn,yn+1=ηνyn+hβνfyn+∑u∈BT∖τhρuρu!αuγuΦνFuyn.

#### A.2. Order Conditions

The algebraic order conditions for the TDRK method with constant coefficients presented in [18] are not applicable for the modified TDRK method (8) whose coefficients depend on *ν* = *ωh*. We expand the exact solution *y*(*t*_*n*_ + *h*) and the numerical solution *y*_*n*+1_ in powers of *h* under the local assumption *y*_*n*_ = *y*(*t*_*n*_):

(A.7) ytn+h=yn+hf+h22!f′f+h33!f′′f,f+f′f′f+⋯,=yn+hf+h22!g+h33!g′f+h44!g′′f,f+g′g+h55!g3f,f,f+3g′′f,g+g′g′f+h66!g4f,f,f,f+6g3f,f,g+3g′′g,g+4g′′f,g′f+g′g′′f,f+g′g′g+⋯,yn+1=ηνyn+hβνf+h2∑i=1sbiνg+g′Yi−yn+12!g′′Yi−yn,Yi−yn+⋯=ηνyn+hβνf+h2bνTeg+h3bνTcg′f+h4bνTAeg′g+12!bνTc2g′′f,f+h5bνTAc·g′g′g+12!bνTc·Ae2g′′f,g+13!bνTc3g3f,f,f+h6bνTA2eg′g′g+12!bνTAc2·g′g′′f,f+13!bνTc2·Ae·3g3f,f,g+14!bνTc4·g4f,f,f,f+⋯,

where *f* and *g* and their partial derivatives are evaluated at *y*_*n*_.

The elementary differentials in the above expressions will be represented geometrically by a set of bicolored (rooted) trees, which are a variation of the rooted trees presented in Butcher [6] and Hairer et al. [8]. For the autonomous system (6), the bicolored trees are made up of two types of vertices: black and white. The first and the simplest tree, which represents *f*, consists of a single black vertex. The time derivative *g* of *f* is expressed by a black vertex connecting upward by a branch to a white vertex . A partial derivative of *g* with respect to *y* is expressed by a white vertex connecting by a branch to a black vertex. Tables 10 and 11 give all the trees of order up to five with the values of the related functions defined on them.

The local truncation error of the modified TDRK method (8) can be expressed in terms of rooted trees which are similar to those defined in Hairer et al. [8]:

(A.8) LEnyn+1−ytn+h=ην−1yn+hβν−1f+∑j=2p+1hj∑ρu=jduFuyn+Ohp+2,

where

(A.9) $$\begin{array}{c} d\left(\;u\;\right)=\frac{1}{j!}\alpha \left(\;u\;\right)\left(\;\gamma \left(\;u\;\right)b{\left(\;\nu \;\right)}^{T}\Phi \left(\;u\;\right)-1\;\right), \end{array}$$

and the order *ρ*(*u*), the density *γ*(*u*), the vector of elementary weights Φ(*u*) = (Φ_1_(*u*),…, Φ_*s*_(*u*))^*T*^, the elementary differential *ℱ*(*u*)(*y*_*n*_) at *y*_*n*_, and the function *α*(*u*) are defined in Appendix A.1. The modified TDRK method (8) has* (algebraic) order p* if its local error satisfies LE_*n*_ = *𝒪*(*h* ^*p*+1^).

The above analysis leads to the order conditions as stated in the following theorem.

Theorem A.8 A.8. The modified TDRK method (8) has (algebraic) order *p*  (*p* ≥ 2) if and only if its coefficients satisfy the following conditions:

(A.10) ην=1+Oνp+1,βν=1+Oνp,bνTΦu=1γu+Oνp−ρu+1,for  all  trees  with  2≤ρu≤p.

Proof The result follows from comparing the expansions of (A.7) and independence of elementary differentials.

#### A.3. The Choice of the Fitting Frequency

It has been known that the choice of the fitting frequency *ω* is crucial to the effectiveness of exponentially or trigonometrically fitted Runge-Kutta methods when applied to initial value problems with oscillatory solutions. In the existing literature, there have been several approaches for estimating the frequency. See, for example, Ixaru and Vanden Berghe [25], Vanden Berghe et al. [26], Ixaru et al. [27], Van de Vyver [28], and Ramos and Vigo-Aguiar [29].

In this paper, we evaluate the “best fitting frequency” *ω* for an EFTDRK method by minimizing the global error. According to (A.8) in Appendix  A.2, we can write the local truncation error of *p*th order modified TDRK method (8) as follows:

(A.11) $$\begin{array}{c} {LE}_{n}=h^{p+1}\underset{u\in T_{p+1}}{\sum }d\left(\;u\;\right)F\left(\;u\;\right)\left(\;y_{n}\;\right)+O\left(\;h^{p+2}\;\right), \end{array}$$

where *T*_*p*+1_ is the set of trees of order *p* + 1. Therefore for small values of step size *h* the global error over the grids *t*_*n*_, *n* = 0,1,…, *N* can be approximated as

(A.12) $$\begin{array}{c} GE\approx h^{p+1}\underset{u\in T_{p+1}}{\sum }d\left(\;u\;\right)\overset{N}{\underset{n=1}{\sum }}F\left(\;u\;\right)\left(\;y_{n}\;\right). \end{array}$$

This shows that the global error depends on *ν* = *hω* and the values of *d*(*u*) and the elementary differentials *ℱ*(*u*) at each *y*_*n*_ and thus depends on the step size *h*, the fitting frequency *ω* of the method, the length of the integration interval, and the function *f* (i.e., the problem to be solved). The “best fitting frequency” can be taken as the minimizer *ω* of the previous approximation of GE.
